# Nomograms for predicting survival in patients with micropapillary bladder cancer: a real-world analysis based on the surveillance, epidemiology, and end results database and external validation in a tertiary center

**DOI:** 10.1186/s12894-023-01183-z

**Published:** 2023-02-13

**Authors:** Peng Liu, Lei Xu, Guanghao Chen, Benkang Shi, Qiujie Zhang, Shouzhen Chen

**Affiliations:** 1 Qilu Hospital of Shandong University, Jinan, China; 2 Department of Geriatrics, Qilu Hospital, College of Medicine, Shandong University, Jinan, China

**Keywords:** Nomogram, Micropapillary bladder cancer, Prognosis, Survival analysis

## Abstract

**Background:**

The present study aimed to construct and validate nomograms that can be used to predict cancer-specific survival (CSS) and overall survival (OS) in patients with micropapillary bladder cancer.

**Methods:**

The data of 627 patients diagnosed with micropapillary bladder cancer between 2000 and 2018 were obtained from the surveillance, epidemiology, and end results database. Patients were randomly divided into the training and internal validation sets (7:3). The Cox proportional hazards regression model was applied to evaluate the association between variables and survival and then nomograms were constructed to predict the survival of an individual patient. The performance of nomograms was validated by using calibration curves, concordance index, receiver operating characteristic curves with the calculated area under the curve and decision curve analysis in the training and internal validation set. Data from 41 micropapillary bladder cancer patients at Qilu Hospital of Shandong University from 2000 to 2022 were collected for external validation.

**Results:**

Several independent risk factors were taken into the two nomograms (CSS and OS), including age, marital status, AJCC TMN stage, surgical approach, lymph node ratio, and tumor size while the OS nomogram additionally contained race. The concordance index of the training set, internal validation set, and external verification set were all over 0.7. The calibration curve indicated good consistence between the nomogram prediction and actual survival. Area under the curve and decision curve analysis results indicated great clinical usefulness of nomograms.

**Conclusions:**

The nomograms predicting the survival outcome of patients with micropapillary bladder cancer would provide a valuable tool to help clinicians to evaluate the risk of patients and make individual treatment strategies.

**Supplementary Information:**

The online version contains supplementary material available at 10.1186/s12894-023-01183-z.

## Background

Micropapillary bladder cancer (MPBC) was first recognized in 1994 by Amin et al. [1], which is characterized by discrete nests of papillary tumors surrounded by lacunae without vascular cores. Micropapillary bladder cancer, a rare variant, accounts for approximately 0.01–2.2% of urothelial carcinoma [2] and a growing body of researches suggest that micropapillary bladder cancer belongs to a subtype of urothelial carcinoma [3]. Unlike conventional bladder cancer, which is mostly non-muscle invasive bladder cancer, micropapillary bladder cancer typically presents with advanced pathological stage at diagnosis and increased risk of metastasis [4, 5]. Numerous studies have confirmed that its prognosis was worse than conventional bladder cancer [6, 7]. However, Richard Naspro et al. found that micropapillary bladder cancer seemed to be weakly associated with reduced survival at radical cystectomy compared to pure urothelial bladder cancer paired for pathologic stage [8]. Given the advantaged stage and controversial prognosis, we urgently need a tool to predict individual prognosis of patients with micropapillary bladder cancer with relative accuracy and then guide enhanced therapies for patients with poor estimated survival outcomes in an effort to improve prognosis.

At present, the prognosis evaluation of the micropapillary bladder cancer patients mainly depends on American Joint Committee for Cancer (AJCC) tumor-node-metastasis (TNM) staging system [9]. The current AJCC staging system only divides patients into different groups and fails to evaluate survival outcomes individually. Due to the failure to consider the demographic and treatment-related information that can affect the prognosis, the prediction of prognosis based on AJCC stage alone is not accurate enough and this prediction model is subject to a lot of skepticism [10–13]. In addition to the above factors, molecular biomarkers and gene expression are also closely related to the prognosis of micropapillary bladder cancer. Steven A Schneider et al. reported that ERBB2 amplification in micropapillary carcinoma could identify patients with poor outcomes [14]. Joep J de Jong et al. found that long non-coding RNAs were associated with aggressive micropapillary-like tumors [15]. However, molecular biomarkers and gene expression data are not always available due to inconvenience and expense. In the real world, clinicians urgently need a convenient and accurate prediction model to assess the prognosis of micropapillary bladder cancer.

Nomograms can simplify the statistical prediction model into a visible tool, which is tailored for single patient and is widely used in the evaluation of cancer prognosis [16]. Nomogram has been applied to predict the prognosis of squamous bladder cancer and small cell carcinoma of the bladder, but no nomogram has been reported on the prognosis of micropapillary bladder cancer. Therefore, we intended to establish validated nomograms based on demographic information (age, sex, race, marital status), clinicopathological parameters (grade, tumor size, TNM stages, positive lymph node ratio) as well as treatment methods (surgery, radiation, chemotherapy) to predict overall survival (OS) and cancer specific survival (CSS) of patients with micropapillary bladder cancer.

## Methods

### Data sources

Data were extracted from SEER using SEER*Stat software version 8.4.0 (http://seer.cancer.gov/) and from Qilu hospital of Shandong University using the hospital’s medical record system. Patient consent and institutional review board approval for the SEER database were not required. The study was performed under the Ethics Committee of Qilu hospital of Shandong University approved protocols, with waiver of written informed consent by patients and in accordance with the Declaration of Helsinki.

### Patient selection

We searched the SEER database including 17 cancer registries and covering 26.5% of the US population. The inclusion site code was C67.0-C67.9, and the histological code was 8131/2, 8131/3, according to the International Classification of Tumor Diseases, Third Edition (ICD-O-3). The exclusion criteria were (1) incomplete survival data; (2) patients in SEER database diagnosed before 2004 since their TNM stage information was not recorded; (3) lymph node examined and positive lymph node were unknown; (4) surgical approach, radiotherapy or chemotherapy was unknown. Patients with micropapillary bladder cancer in Qilu hospital of Shandong University were considered to be included in the external validation set. The exclusion criteria were similar to those of the SEER database. After selection, 627 patients were enrolled from the SEER database and 41 patients from the tertiary center.

### Variables

The information collected included age, sex, race, annual income, marital status, histologic type, grade, American Joint Committee on Cancer stage I-IV, AJCC T stage, AJCC N stage, AJCC M stage, surgery of primary site, lymph node examined and positive lymph node, radiotherapy, chemotherapy, tumor size, survival months and status. Age was regrouped into “< 80” and “>  = 80”, annual income was regrouped into “< $60,000” and “>  = $60,000”. Never married, Widowed, Separated and Divorced were classified as Single and the AJCC stage I^b^ included I, 0a and 0is. In terms of AJCC N stage, N1, N2, and N3 were all classified as N1. The surgical treatment variable was grouped into “Non-complete cystectomy” (RX Summ-Surg Prim Site code10-30), “Radical cystectomy” (RX Summ-Surg Prim Site code 50–80) and “No surgery” (RX Summ-Surg Prim Site code 00). Tumor size, and lymph node ratio (positive lymph node / lymph node examined) were turned to categorical variable using receiver operating characteristic curve (ROC).

### Outcomes

The primary outcome was CSS, defined as the time from the first diagnosis of micropapillary bladder cancer to cancer-specific death, and the secondary outcome was OS, defined as the time from the first diagnosis to death from any cause.

### Construction and validation of the nomogram

Eligible micropapillary bladder cancer patients from the SEER database were randomly divided into a training set and an internal validation set using a ratio of 7:3. Micropapillary bladder cancer patients from the tertiary center were designated as the external validation set. External validation was performed to further verify the accuracy of the nomograms.

Univariate and multivariate Cox regression analyses were performed on patients in the training set to evaluate the variable’s impact on CSS and OS, presenting as a hazard ratio (HR) and corresponding 95% confidence interval (CI). Based on results of the multivariate Cox regression analysis, nomograms were created for predicting the probability of 1-, 3-, and 5-year CSS and OS. The internal validation of the nomogram was performed in the internal validation set and the external validation was conducted using external validation set. Validation of this nomogram was then performed using bootstrapping with 1000 resamples. The accuracy of nomograms was evaluated using a calibration curve through comparing nomogram-predicted survival with actual survival. The discriminatory ability of the nomogram was evaluated by the concordance index (C-index) and the receiver operating characteristic (ROC) curves with the calculated area under the curve (AUC). In addition, decision curve analysis (DCA) was applied to estimate the clinical net benefit of the nomogram by comparing the threshold probabilities range of the model to that of the AJCC staging system.

All statistical analyses were conducted by R version 4.2.0 (http://www.R-project.org), and a P-value < 0.05 was considered significant.

## Results

### Patients baseline characters

**Fig. 1:**
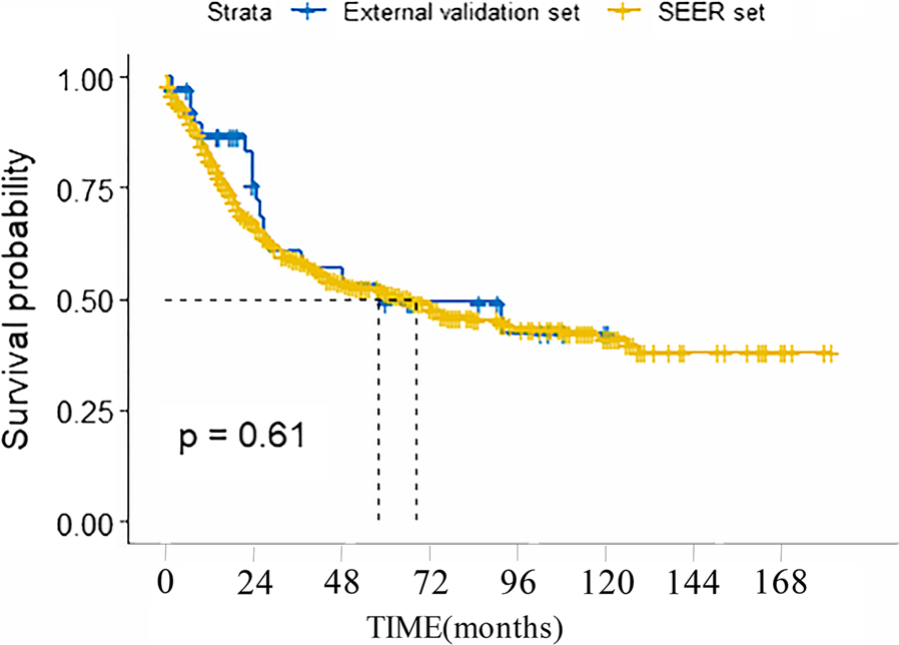
The cancer-specific survival of micropapillary bladder cancer

According to the inclusion criteria, a total of 668 micropapillary patients were retrospectively enrolled from SEER (n = 627) and tertiary centers (n = 41). By the end of follow-up, a total of 360 of the 627 people enrolled from SEER database had died, of which 280 died from micropapillary bladder cancer and the remaining 80 died from other causes. Details on the demographic information, clinicopathological parameters, and treatments in the training set, internal validation set, and external validation set were shown in Table 1. There was no substantive difference in various indicators between training set and internal validation set. There were statistical differences between SEER set and external validation set in race, marital status, AJCC stage, histological grade and chemotherapy (*p* < 0.05). In the SEER set, most of the patients were less than 80 years old (77.83%), and most of the patients were white (89.63%), the majority histological grade was G3 and G4 (70.69%). Median follow-up period was 21 months (interquartile range from 9.0 to 54.5 months). The 1-, 3-, and 5-year CSS rate was 81.6%, 62.0% and 53.2% respectively in the SEER set. In the external validation set, at the last follow-up, 16 patients died of a cancer-specific cause, and 2 patients died from other causes; the median follow-up time was 24 months (interquartile range from 12.0 to 66.0 months). The 1-, 3-, and 5-year CSS rate was 87.6%, 62.8% and 51.2% respectively (Fig. 1).

**Table 1:** Characteristics of patients

	SEER set (n = 627)	Training set (n = 435)	Internal volidation set (n = 192)	*p* value	Enternal Volidation set (n = 41)	*p* value
Age (year), n (%)						
< 80	488 (77.83)	346 (79.54)	142 (73.96)	0.121	34 (82.93)	0.444
> = 80	139 (22.17)	89 (20.46)	50 (26.04)		7 (17.07)	
Race, n (%)						
Black	34 (5.42)	28 (6.44)	6 (3.12)	0.190	0 (0.00)	0.001
White	562 (89.63)	384 (88.28)	178 (92.71)		0 (0.00)	
Other^a^	31 (4.94)	23 (5.29)	8 (4.17)		41 (100.00)	
Sex, n (%)						
Male	506 (80.70)	346 (79.54)	160 (83.33)	0.267	39 (95.12)	0.021
Female	121 (19.30)	89 (20.46)	32 (16.67)		2 (4.88)	
Marital status, n (%)						
Married	396 (63.16)	274 (62.99)	122 (63.54)	0.791	38 (92.68)	0.001
Single	200 (31.90)	141 (32.41)	59 (30.73)		3 (7.32)	
Unknown	31 (4.94)	20 (4.60)	11 (5.73)		0 (0.00)	
AJCC stage, n (%)						
I^b^	209 (33.33)	149 (34.25)	60 (31.25)	0.233	14 (34.15)	0.001
II	168 (26.79)	107 (24.60)	61 (31.77)		8 (19.51)	
III	81 (12.92)	61 (14.02)	20 (10.42)		14 (34.15)	
IV	169 (26.95)	118 (27.13)	51 (26.56)		5 (12.19)	
AJCC T, n (%)						
Ta	18 (2.87)	13 (2.99)	5 (2.60)	0.300	1 (2.44)	0.973
Tis	6 (0.96)	5 (1.15)	1 (0.52)		0 (0.00)	
T1	196 (31.26)	138 (31.72)	58 (30.21)		15 (36.58)	
T2	232 (37.00)	171 (39.31)	61 (31.77)		14 (34.15)	
T3	94 (14.99)	60 (13.79)	34 (17.71)		6 (14.63)	
T4	81 (12.92)	48 (11.03)	33 (17.19)		5 (12.20)	
AJCC N, n (%)						
N0	458 (73.05)	316 (72.64)	142 (73.96)	0.732	27 (65.85)	0.317
N1-3	169 (26.95)	119 (27.36)	50 (26.04)		14 (34.15)	
AJCC M, n (%)						
M0	563 (89.79)	389 (89.43)	174 (90.62)	0.647	37 (90.24)	0.926
M1	64 (10.21)	46 (10.57)	18 (9.38)		4 (9.76)	
Grade						
I	8 (1.28)	7 (1.61)	1 (0.52)	0.752	0 (0.00)	0.001
II	15 (2.39)	11 (2.53)	4 (2.08)		0 (0.00)	
III	94 (14.99)	67 (15.40)	27 (14.06)		0 (0.00)	
IV	349 (55.66)	237 (54.48)	112 (58.33)		0 (0.00)	
Unknown	161 (25.68)	113 (25.98)	48 (25.00)		41 (100.00)	
Surgery						
None	17 (2.71)	10 (2.30)	7 (3.65)	0.430	1 (2.44)	0.086
Local excision	353 (56.30)	241 (55.40)	112 (58.33)		16 (39.02)	
Complete cystectomy	257 (40.99)	184 (42.30)	73 (38.02)		24 (58.54)	
Lymph node ratio						
None, Biopsy	372 (59.33)	254 (58.39)	118 (61.46)	0.590	21 (51.22)	0.567
< 0.061	123 (19.62)	90 (20.69)	33 (17.19)		9 (21.95)	
≥ 0.061	132 (21.05)	91 (20.92)	41 (21.35)		11 (26.83)	
Radiotherapy						
Yes	70 (11.16)	44 (10.11)	26 (13.54)	0.209	4 (9.76)	0.781
No	557 (88.84)	391 (89.89)	166 (86.46)		37 (90.24)	
Chemotherapy						
Yes	298 (47.53)	206 (47.36)	92 (47.92)	0.897	10 (24.39)	0.004
No	329 (52.47)	229 (52.64)	100 (52.08)		31 (75.61)	
Tumor size (mm)						
≤ 31.5	173 (27.59)	126 (28.97)	47 (24.48)	0.508	11 (26.83)	0.990
> 31.5	208 (33.18)	141 (32.41)	67 (34.90)		14 (34.15)	
Unknown	246 (39.23)	168 (38.62)	78 (40.62)		16 (39.02)	

Other^a^ comprises American Indian/Alaska Native, Asian/Pacific Islander

I^b^ comprises AJCC stage 0a, 0is, I

### Univariable and multivariable cox regression in the training set

In univariable Cox regression analysis for CSS, eleven factors (age, sex, histologic grade, marital status, T stage, N stage, M stage, AJCC TMN stage, surgical approach, radiotherapy, lymph node ratio, and tumor size) reached statistical significance. Then, above eleven factors were included in the multivariate Cox analysis. The variables with *p* < 0.05 were identified as independent prognostic factors, including age, marital status, AJCC TMN stage, surgical approach, lymph node ratio, and tumor size (Table 2).

**Table 2:** Univariable and Multivariable Analyses of cancer-specific survival in the Ttraining set

Subject characteristics	Univariate		Multivariate	
	HR (95% CI)	*p* value	HR (95% CI)	*p* value
Age (year), n (%)				
< 80	1 (reference)	1.000	1 (reference)	1.000
> = 80	1.81 (1.27–2.58)	0.001	2.62 (1.80, 3.82)	< 0.001
Race, n (%)				
Black	1 (reference)	1.000	1 (reference)	
White	0.70 (0.40–1.24)	0.223	–	–
Other^a^	0.40 (0.15–1.06)	0.066	–	–
Sex, n (%)				
Female	1 (reference)	1.000	1 (reference)	1.000
Male	0.70 (0.50–1.00)	0.047	0.90 (0.62, 1.29)	0.561
Marital status, n (%)				
Married	1 (reference)	1.000	1 (reference)	1.000
Single	1.52 (1.11–2.07)	0.009	1.60 (1.14, 2.23)	0.006
Unknown	1.01 (0.47–2.17)	0.980	1.46 (0.66, 3.26)	0.351
Income				
< $60,000	1 (reference)	1.000	1 (reference)	
> = $60,000	0.96 (0.70–1.33)	0.824	–	–
AJCC stage, n (%)				
I^b^	1 (reference)	1.000	1 (reference)	1.000
II	2.83 (1.75–4.59)	< 0.001	2.08 (0.62, 7.04)	0.238
III	3.23 (1.85–5.64)	< 0.001	3.48 (1.00, 12.13)	0.050
IV	6.72 (4.36–10.35)	< 0.001	5.28 (1.51, 18.46)	0.009
AJCC T, n (%)				
Ta	0.25 (0.03–1.81)	0.250	0.17 (0.02, 1.31)	0.090
Tis	1.5 (0.36–6.26)	0.582	1.95 (0.46, 8.34)	0.367
T1	1 (reference)	1.000	1 (reference)	1.000
T2	2.93 (1.89–4.56)	< 0.001	1.69 (0.56, 5.09)	0.353
T3	3.95 (2.44–6.40)	< 0.001	1.79 (0.57, 5.58)	0.318
T4	5.4 (3.33–8.75)	< 0.001	1.34 (0.44, 4.13)	0.609
AJCC N, n (%)				
N0	1 (reference)	1.000	1 (reference)	1.000
N1-3	3.05 (2.24–4.15)	< 0.001	0.58 (0.29, 1.15)	0.120
AJCC M, n (%)				
M0	1 (reference)	1.000	1 (reference)	1.000
M1	3.92 (2.69–5.71)	< 0.001	1.49 (0.88, 2.52)	0.135
Grade				
I	1 (reference)	1.000	1 (reference)	
II	1.46 (0.13–16.10)	0.757	–	–
III	5.45 (0.74–39.93)	0.095	–	–
IV	6.01 (0.84–43.18)	0.075	–	–
Unknown	6.46 (0.88–47.65)	0.067	–	–
Surgery				
None	1 (reference)	1.000	1 (reference)	1.000
Local excision	0.22 (0.10–0.47)	< 0.001	0.39 (0.16, 0.96)	0.042
Complete cystectomy	0.29 (0.13–0.63)	0.002	0.33 (0.13, 0.86)	0.024
Lymph node ratio				
None, Biopsy	1.88 (1.17–3.03)	0.009	2.59 (1.31, 5.12)	0.006
< 0.061	1 (reference)	1.000	1 (reference)	1.000
≥ 0.061	5.22 (3.16–8.63)	< 0.001	4.13 (1.95, 8.74)	< 0.001
Radiotherapy				
No	1 (reference)	1.000	1 (reference)	1.000
Yes	2.21 (1.44–3.4)	< 0.001	0.96 (0.58, 1.58)	0.863
Chemotherapy				
No	1 (reference)	1.000	1 (reference)	
Yes	1.14 (0.85–1.54)	0.385	–	–
Tumor size (mm)				
≤ 31.5	1 (reference)	1.000	1 (reference)	1.000
> 31.5	2.12 (1.41–3.19)	< 0.001	1.73 (1.12, 2.69)	0.014
None/Unknown	1.57 (1.05–2.35)	0.027	1.49 (0.97, 2.29)	0.066

Other^a^ comprises American Indian/Alaska Native, Asian/Pacific Islander

I^b^ comprises AJCC stage 0a, 0is, I

In univariable Cox regression analysis for OS, twelve factors (age, race, histologic grade, marital status, T stage, N stage, M stage, AJCC TMN stage, surgical approach, radiotherapy, lymph node ratio, and tumor size) reached statistical significance. Then, above twelve factors were included in the multivariate Cox analysis. The variables with *p* < 0.05 were identified as independent prognostic factors, including age, race, marital status, AJCC TMN stage, surgical approach, lymph node ratio, and tumor size (Additional file 1: Table S1).

#### Nomogram construction

**Fig. 2:**
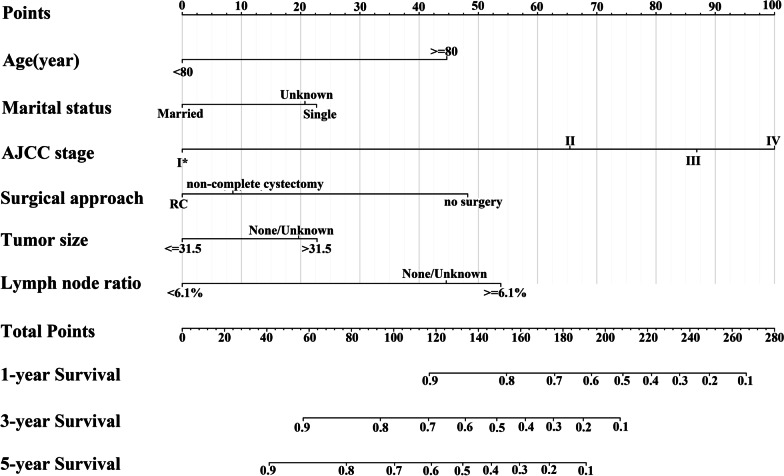
Nomograms predicting the 1-, 3-, 5-year CSS of micropapillary bladder cancer. I ^*^ comprises AJCC stage 0a, 0is, I. CSS, cancer specific survival; AJCC, American Joint Commission on Cancer; RC: radical cystectomy

Nomograms were established based on the above independent prognostic factors for predicting the 1-, 3-, 5-year CSS and OS (Fig. 2, Additional file 1: Fig. S1).

Each of the independent prognostic factors was given a point according to HR. The scores corresponding to independent prognostic factors were added to obtain the total score which is located onto the total points scale to obtain the probability of 1-, 3-, and 5- year CSS and OS. Nomograms can make individualized prediction based on patient information, improving the accuracy and efficiency of prediction. For example, if a 70-year old single patient was found no positive lymph node metastasis, the tumor size was 4 cm, and the AJCC stage was III after radical cystectomy, he would score 130 points, which means that this patient has approximately 86% possibility of cancer-special survival in the first year, approximately 62% possibility of cancer-special survival in the third year and approximately 52% possibility of cancer-special survival in the fifth year.

#### Nomogram validation

##### Internal validation

Nomograms were validated internally in the training set and the internal validation set.

In the training set, C-indices of the nomogram were 0.766 (95% CI 0.735–0.797), 0.742 (95% CI 0.726–0.758) for CSS and OS respectively, which were both higher than 0.7, suggesting that these two nomograms were relatively accurate and suitable for predicting CSS and OS for patients with micropapillary bladder cancer. In the internal validation set, C-indices of the nomogram were 0.753 (95% CI 0.699–0.808), and 0.738 (95% CI 0.715–0.761) for CSS and OS respectively.

**Fig. 3:**
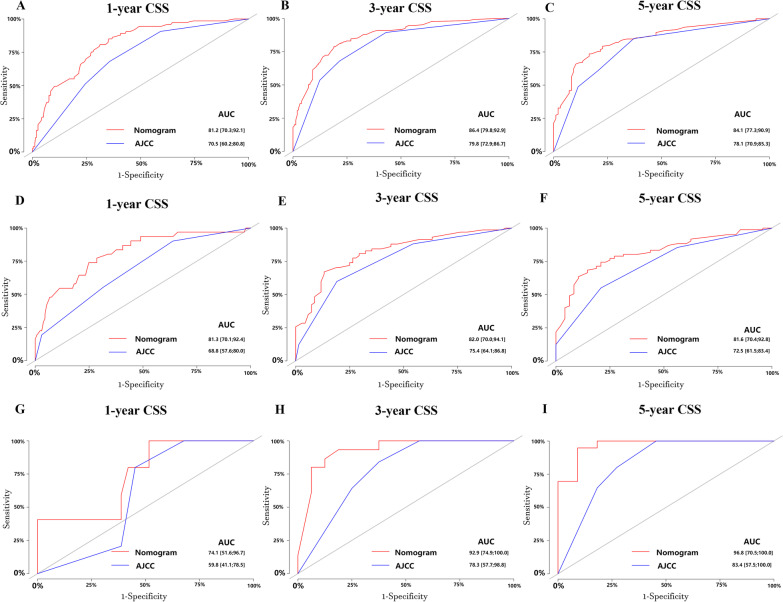
ROC curves in the training set (**A**–**C**), internal validation set (**D**–**F**), external validation set (**G**–**I**). CSS, cancer specific survival; AJCC, American Joint Commission on Cancer

For micropapillary bladder cancer patients, the 1-, 3-, and 5-year CSS of the nomogram yielded AUC values of 0.812, 0.864, and 0.841 in the training set (Fig. 3A–C), 0.813, 0.820, 0.816 in the internal validation set (Fig. 3D–F). And the 1-, 3-, and 5-year OS of the nomogram yielded AUC values of 0.788, 0.832, and 0.829 in the training set, 0.807, 0.800, 0.801 in the internal validation set (Additional file 1: Fig. S2). AUC values of our model were larger than AJCC stage, indicating that nomograms showed better discrimination.

**Fig. 4:**
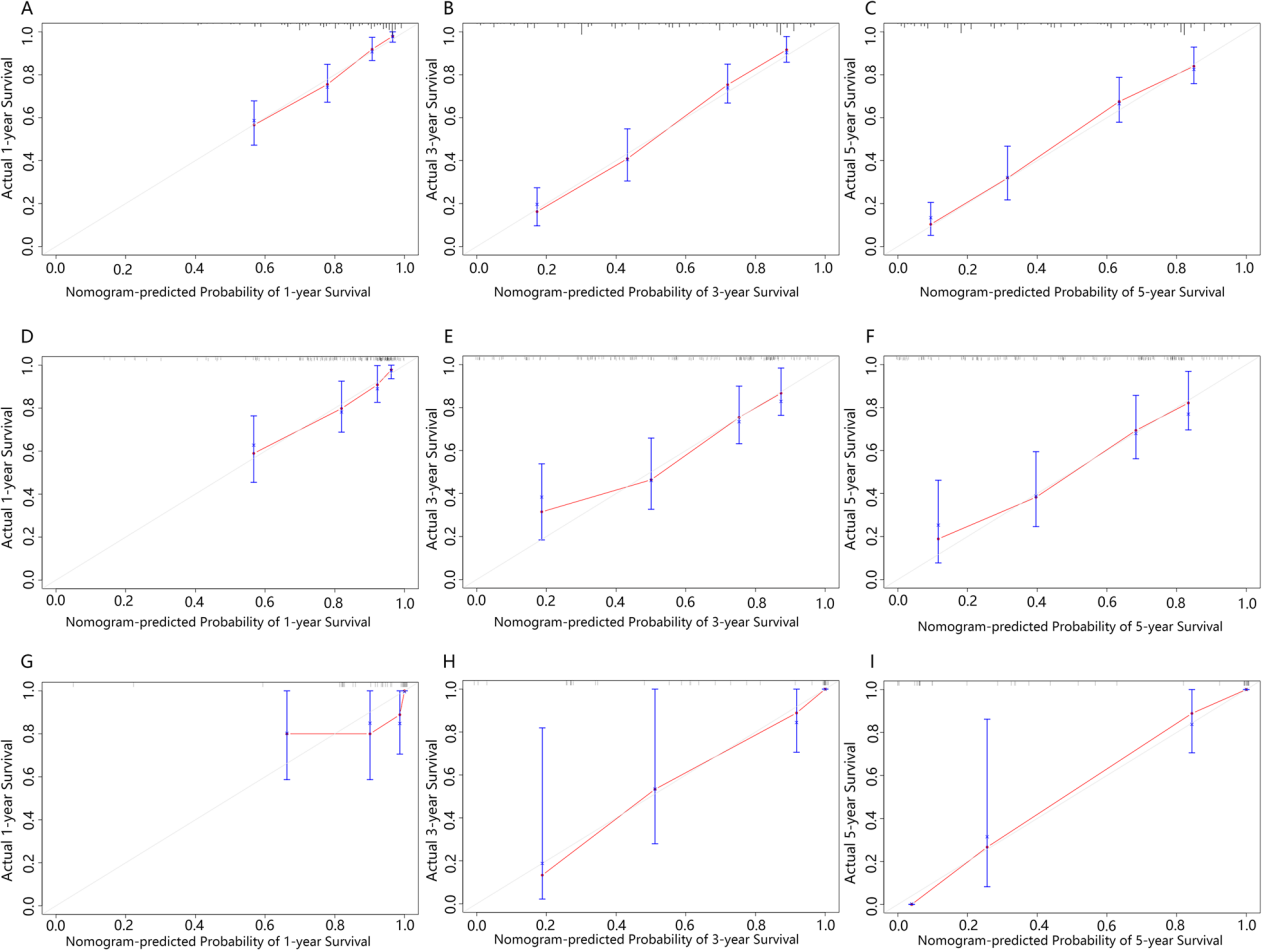
Calibration curves in the training set (**A**–**C**), internal validation set (**D**–**F**), external validation set (**G**–**I**). Nomogram-predicted probability of survival was plotted on the X-axis, and the actual probability of survival was plotted on the Y-axis. The perfect calibration model was represented by dashed lines which indicated actual probability was exactly the same as predicted probability. The distance between solid lines and dashed lines represented the fitness of actual and nomogram-predicted prognosis. Abbreviations: CSS, cancer specific survival

The calibration curves of the nomogram were highly consistent with the standard curve, which indicated that the nomogram showed high reliability in predicting 1-, 3-, and 5-year CSS in the training (Fig. 4A–C) and internal validation sets (Fig. 4D–F) as well as 1-, 3-, and 5-year OS in the training and internal validation sets (Additional file 1: Fig. S3).

**Fig. 5:**
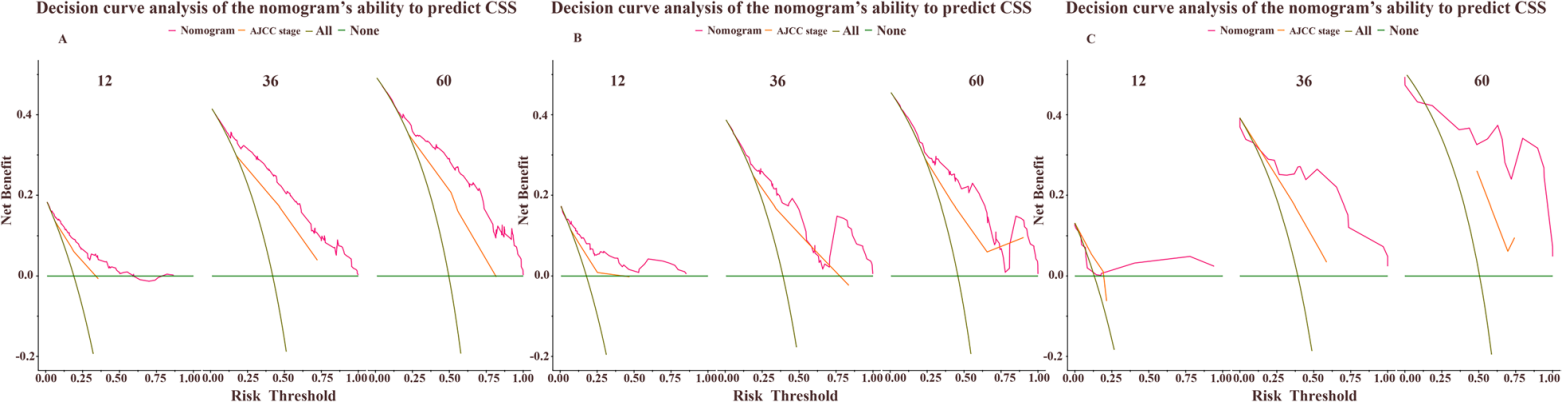
DCA curves in the training set (**A**), internal validation set (**B**), external validation set (**C**). CSS, cancer specific survival; AJCC, American Joint Commission on Cancer

DCA of the nomograms showed higher net benefits and demonstrated better clinical outcome values than those obtained using AJCC stage for CSS in the training (Fig. 5A), internal validation (Fig. 5B) as well as OS (Additional file 1: Fig. S4).

#### External validation

In the external validation set, C-indices of the nomograms were 0.813, 0.828 for CSS and OS respectively. The AUC values were 0.741, 0.929, and 0.968 for the 1-, 3-, and 5-year CSS (Fig. 3G–I), 0.763, 0.960, and 0.990 for the 1-, 3-, and 5-year OS (Additional file 1: Fig. S2). The calibration curves of the nomogram were highly consistent with the standard curve in predicting 1-, 3-, and 5-year CSS (Fig. 4G–I) as well as 1-, 3-, and 5-year OS (Additional file 1: Fig. S3). DCA of the nomograms showed higher net benefits and demonstrated better clinical outcome values than those obtained using AJCC stage for CSS (Fig. 5C) and OS (Additional file 1: Fig. S4). Some of the DCA curves in the external validation set were incomplete, which relates to the small sample size of the external validation set. In particular, the sample size of patients with 5-year survival data was more limited.

## Discussion

Micropapillary carcinoma, a rare variant, is composed of infiltrating slender delicate filiform processes or small tight papillary tumor cell clusters that lie within lacunae [17]. Micropapillary bladder cancer is characterized by advanced pathological stage at diagnosis and prone to lymph node metastasis, so the prognosis is very poor [2, 18]. The proportion of micropapillary component has been extensively studied for its effect on prognosis and is now considered that clinical significance is associated with even a small amount of micropapillary histology relative to conventional urothelial carcinoma [19, 20]. Because of its rarity and lack of clear guideline, clinicians have a poor understanding of treatment and prognosis of micropapillary bladder cancer.

Although AJCC TNM staging system is popular for cancer prognosis, the rationality of its application in micropapillary bladder cancer has been much debated. Firstly, the TNM staging system is established mainly based on conventional urothelial carcinoma instead of micropapillary bladder cancer. Micropapillary bladder cancer has been reported to have a poorer prognosis than conventional bladder cancer, but a recent study showed that clinical outcomes were comparable to conventional bladder cancer after controlling for standard clinicopathologic predictors [2]. Currently, there is no suitable tool to access prognosis of patients with micropapillary bladder cancer individually. Besides, the TNM system groups patients only depended on pathological features, without considering demographic and treatment-related information, which reduced the accuracy of prediction. In the real world, clinicians urgently need a convenient and accurate prediction model to evaluate individual patient survival outcomes of micropapillary bladder cancer. Nomograms, as a visual tool, are widely used for cancer prognosis, primarily because of their ability to reduce statistical predictive models into a single numerical estimate of the probability of an event, that is tailored to the profile of an individual patient [16]. However, there is no reliable, large sample-based, real-world tool for evaluating postoperative prognosis among adult patients with micropapillary bladder cancer. Therefore, the two prognostic nomograms for micropapillary bladder cancer patients established using data from the SEER database in this study should be quite valuable and practical for clinicians.

Our study identified six independent prognostic factors for CSS, including age, marital status, AJCC stage, tumor size, surgery approach and positive lymph node ratio.

Generally, older patients with decreased immunity and increased numbers of comorbidities are more likely to have poorer survival outcomes. Although age has not been reported to affect the prognosis of micropapillary bladder cancer, its effect on conventional bladder cancer and micropapillary breast cancer has been widely concerned [21–23]. Gary D Lewis et al. conducted a retrospective analysis of 2,660 patients with invasive micropapillary carcinoma, reporting that age < 65 years were associated with prolonged OS [23]. In the present study, age ≥ 80 years was an independent prognostic factor for CSS in patients with micropapillary bladder cancer after surgery, and the results were basically the same.

Marital status was used as a predictor of survival for various tumors, including bladder cancer [24–26]. Numerous studies had reported that married patients tend to have better survival outcomes, which is consistent to our nomograms. There could be several reasons. First of all, married patients may get more attention from their partners or children, which will greatly affect the patients' mentality and quality of life [27]. What's more, married patients tend to be better off financially and have greater access to treatment resources.

Similar to other tumors, clinicopathological features are also important prognostic indicators of micropapillary bladder cancer. Z Li et al. reported that micropapillary bladder cancer often presented at an advanced stage with lymphovascular invasion and distant metastases, and the prognosis was poor [18]. Richard Naspro et al. found that patients with lymph node metastasis and advanced T stage had worse survival outcomes [28]. Mario I Fernández et al. conducted a retrospective analysis of 103 micropapillary bladder cancer patients undergoing radical cystectomy, reporting that the 5-year disease-specific survival for patients with T1, >  = T2 were 92%, 51% respectively (*p* < 0.001).

In terms of surgical treatment, radical cystectomy is considered as the gold standard for muscle-invasive bladder cancer and refractory non-muscle-invasive bladder cancer. The optimal treatment of patients with cT1 remains controversial [12]. Given the pathologic characteristics of micropapillary bladder cancer, implementation of early radical cystectomy in patients with cT1 is recommended. Mohammad Abufaraj et al. conducted a systemic review and found that radical cystectomy was associated with a better prognosis [12]. In other words, previous studies have shown that adding these variables to our nomograms will help improve accuracy. Moreover, from ROC and DCA in this study, the nomogram model has a significantly higher prognostic accuracy than the AJCC stage system. To the best of our knowledge, this is the first nomogram established to predict the survival of micropapillary bladder cancer patients using the SEER database, and we validated it with an external set with long-term follow-up.

However, there were several limitations in our study. First, this study was a retrospective analysis with inherent biases. Further efforts are needed to collect prospective data. Second, some important information is unavailable in the SEER database, such as blood test information, smoking history. Comorbidities information is also an important prognostic indicator, which can affect treatment choices and survival. Lack of comorbidity information is one of the limitations of this paper. Furthermore, although the C-indices of the two nomograms were greater than 0.7, indicating high accuracy in CSS and OS, about 30% of our predictions were wrong and nomograms need to be further improved.

## Conclusion

We established the visible nomograms to predict individual CSS and OS of micropapillary bladder cancer patients using demographic information, clinicopathological factors and treatment-related factors. The internal validation and external validation of the model proved its significant performance. The nomograms can be valuable in assisting patient counseling and guiding treatment decision making in areas such as prognostic evaluation, individualized therapy, and clinical trial design.

## Supplementary Information

**Additional file 1:** Construction and validation of nomogram for predicting overall survival (OS) in patients with micropapillary bladder cancer.

## Data Availability

The dataset supporting the conclusions of this article is available in the SEER*Stat software version 8.4.0 repository (http://seer.cancer.gov/). Other datasets will be available from the corresponding author on reasonable request.

## Abbreviations

CSS: Cancer-specific survival
OS: Overall survival
SEER: Surveillance, epidemiology, and end results
ROC: Receiver operating characteristic
AUC: Area under the curve
DCA: Decision curve analysis
C-index: Concordance index
AJCC: American Joint Committee for Cancer
TNM: Tumor-node-metastasis
MPUC: Micropapillary bladder cancer
HR: Hazard ratio
CI: Confidence interval
