# Supplementary material for: Nomograms for predicting survival in patients with micropapillary bladder cancer: a real-world analysis based on the surveillance, epidemiology, and end results database and external validation in a tertiary center
Source: BMC Urol. 2023 Feb 13;23:16. doi: 10.1186/s12894-023-01183-z (PMC9926703; doi:10.1186/s12894-023-01183-z)
Supplement: Supplementary file 1 — Additional file 1: Construction and validation of nomogram for predicting overall survival (OS) in patients with micropapillary bladder cancer. [file 12894_2023_1183_MOESM1_ESM.docx]

**Supplemental Table S1 Univariable and Multivariable Analyses of Overall Survival in the Ttraining Cohrot**

| Subject  characteristics | Univariate |  |  | Multivariate |  |
| --- | --- | --- | --- | --- | --- |
|  | **HR(95% CI)** | ***P* value** |  | **HR(95% CI)** | ***P* value** |
| **Age (year), n (%)** |  |  |  |  |  |
| <80 | 1 (reference) | 1.00 |  | 1 (reference) | 1.00 |
| >=80 | 1.9 (1.22-2.94) | **0.004** |  | 2.87 (2.11, 3.90) | **0.001** |
| **Race, n (%)** |  |  |  |  |  |
| Black | 1 (reference) | 1.00 |  | 1 (reference) | 1.00 |
| White | 0.67 (0.42-1.06) | 0.088 |  | 0.61 (0.36, 1.01) | 0.055 |
| Other^1^ | 0.47 (0.22-1) | **0.049** |  | 0.40 (0.18, 0.90) | **0.027** |
| **Sex, n (%)** |  |  |  |  |  |
| Female | 1 (reference) | 1.00 |  | 1 (reference) |  |
| Male | 0.82 (0.61-1.12) | 0.211 |  | - | - |
| **Marital status, n (%)** |  |  |  |  |  |
| Married | 1 (reference) | 1.00 |  | 1 (reference) | 1.00 |
| Single | 1.41 (1.08-1.83) | **0.011** |  | 1.48 (1.11, 1.97) | **0.008** |
| Unknown | 0.79 (0.4-1.55) | 0.494 |  | 0.92 (0.46, 1.84) | 0.806 |
| **Income** |  |  |  |  |  |
| <$60,000 | 1 (reference) | 1.00 |  | 1 (reference) |  |
| >=$60,000 | 0.89 (0.69-1.17) | 0.412 |  | - | - |
| **AJCC stage, n (%)** |  |  |  |  |  |
| I ^2^ | 1 (reference) | 1.00 |  | 1 (reference) | 1.00 |
| II | 2.35 (1.63-3.38) | **<0.001** |  | 2.00 (0.74, 5.43) | 0.175 |
| III | 2.34 (1.5-3.64) | **<0.001** |  | 2.40 (0.87, 6.64) | 0.091 |
| IV | 4.3 (3.07-6.02) | **<0.001** |  | 3.29 (1.17, 9.22) | **0.024** |
| **AJCC T, n (%)** |  |  |  |  |  |
| T1 | 1 (reference) | 1.00 |  | 1 (reference) | 1.00 |
| Ta | 0.84 (0.36-1.94) | 0.677 |  | 0.66 (0.28, 1.55) | 0.336 |
| Tis | 1.27 (0.4-4.05) | 0.692 |  | 1.42 (0.44, 4.60) | 0.559 |
| T2 | 2.45 (1.74-3.45) | **<0.001** |  | 1.51 (0.60, 3.77) | 0.381 |
| T3 | 2.73 (1.83-4.06) | **<0.001** |  | 1.65 (0.64, 4.21) | 0.298 |
| T4 | 3.98 (2.69-5.91) | **<0.001** |  | 1.37 (0.55, 3.45) | 0.501 |
| **AJCC N, n (%)** |  |  |  |  |  |
| N0 | 1 (reference) | 1.00 |  | 1 (reference) | 1.00 |
| N1-3 | 2.29 (1.76-2.99) | **<0.001** |  | 0.64 (0.35, 1.16) | 0.140 |
| **AJCC M, n (%)** |  |  |  |  |  |
| M0 | 1 (reference) | 1.00 |  | 1 (reference) | 1.00 |
| M1 | 2.95 (2.09-4.16) | **<0.001** |  | 1.31 (0.82, 2.10) | 0.26 |
| **Grade** |  |  |  |  |  |
| I | 1 (reference) | 1.00 |  | 1 (reference) |  |
| II | 1.99 (0.38-10.25) | 0.413 |  | - | - |
| III | 4.1 (0.99-17.04) | 0.052 |  | - | - |
| IV | 5.42 (1.33-22.07) | **0.018** |  | - | - |
| Unknown | 5.57 (1.33-23.28) | **0.019** |  | - | - |
| **Surgery** |  |  |  |  |  |
| None | 1 (reference) | 1.00 |  | 1 (reference) | 1.00 |
| Local excision | 0.26 (0.13-0.51) | **<0.001** |  | 0.40 (0.18, 0.85) | **0.018** |
| Complete cystectomy | 0.32 (0.16-0.63) | **0.001** |  | 0.40 (0.17, 0.94) | **0.034** |
| **Lymph node ratio** |  |  |  |  |  |
| ＜0.061 | 1 (reference) | 1.00 |  | 1 (reference) | 1.00 |
| ≥0.061 | 3.93 (2.59-5.96) | **<0.001** |  | 3.51 (1.89, 6.50) | **<0.001** |
| None, Biopsy | 1.83 (1.25-2.67) | **0.002** |  | 2.75 (1.58, 4.78) | **<0.001** |
| **Radiotherapy** |  |  |  |  |  |
| No | 1 (reference) | 1.00 |  | 1 (reference) | 1.00 |
| Yes | 1.93 (1.32-2.83) | **<0.001** |  | 0.93 (0.59, 1.44) | 0.731 |
| **Chemotherapy** |  |  |  |  |  |
| No | 1 (reference) | 1.00 |  | 1 (reference) |  |
| Yes | 0.99 (0.76-1.27) | 0.919 |  | - | - |
| **Tumor size (mm)** |  |  |  |  |  |
| ≤31.5 | 1 (reference) | 1.00 |  | 1 (reference) | 1.00 |
| ＞31.5 | 1.87 (1.33-2.62) | **<0.001** |  | 1.63 (1.14, 2.34) | **0.008** |
| None/Unknown | 1.53 (1.1-2.11) | **0.011** |  | 1.43 (1.01, 2.01) | **0.045** |
|  | **Note: other^1^ comprises American Indian/Alaska Native, Asian/Pacific Islander. I ^2^ comprises AJCC stage 0a, 0is, I** | | | | |

**Supplemental Figure S1** Nomograms predicting the 1-, 3-, 5-year OS of micropapillary bladder cancer


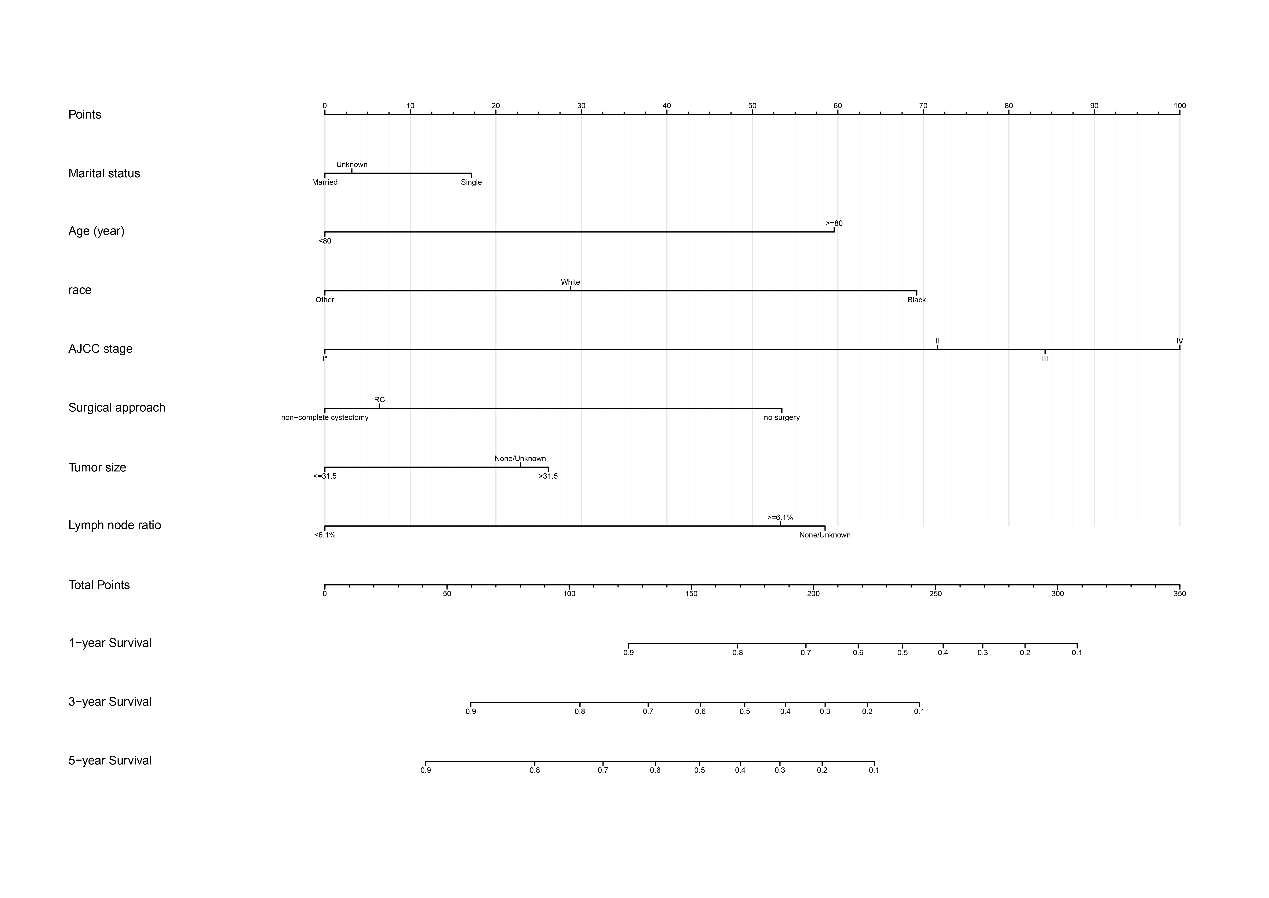


I ^*^ comprises AJCC stage 0a, 0is, I. Abbreviations: OS, overall specific survival; AJCC, American Joint Commission on Cancer; RC: radical cystectomy.

**Supplemental Figure S2** ROC curves in the training set (A-C), internal validation set (D-F), external validation set (G-I).


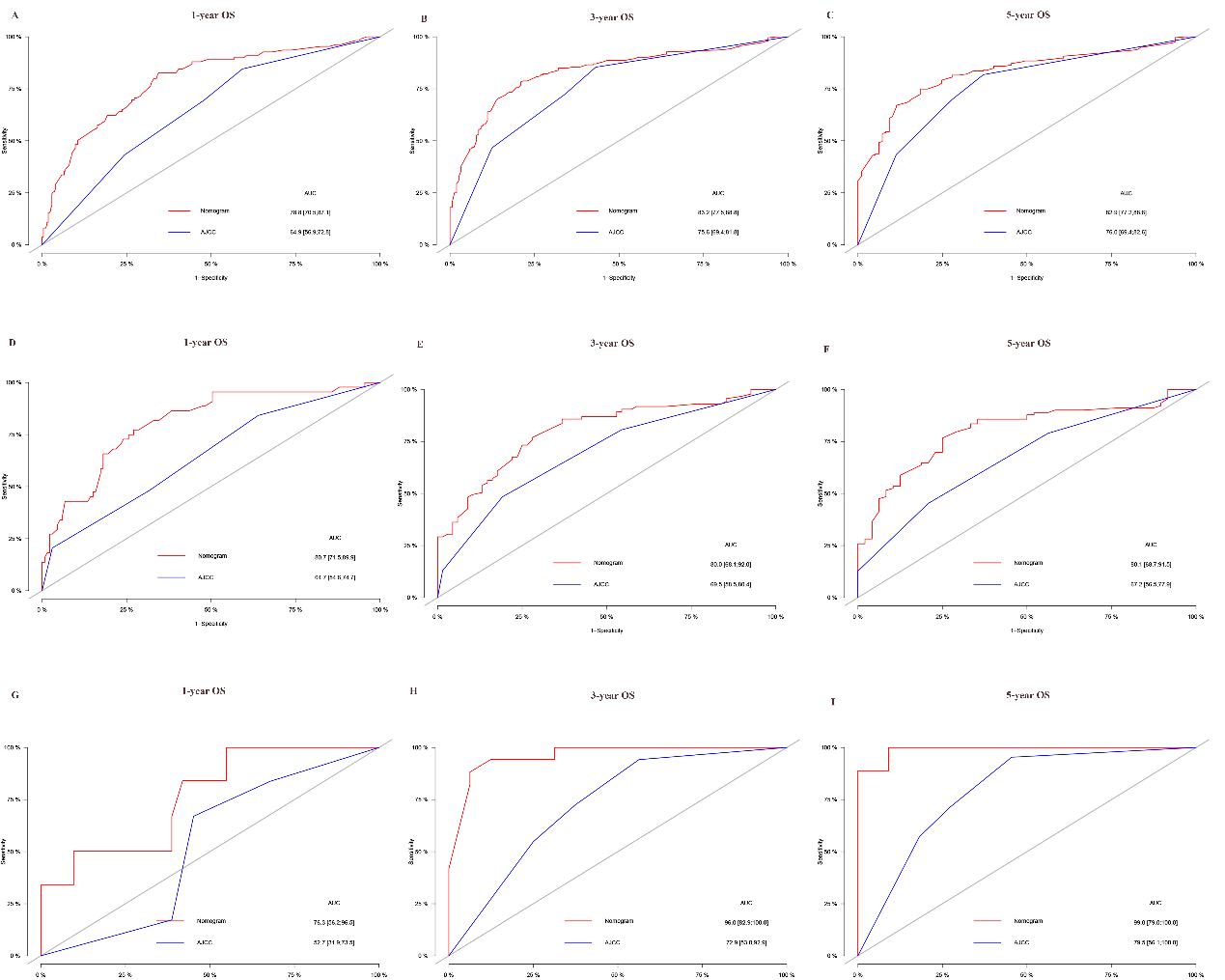


Abbreviations: OS, overall survival; AJCC, American Joint Commission on Cancer.

**Supplemental Figure S3** Calibration curves in the training set (A-C), internal validation set (D-F), external validation set (G-I).


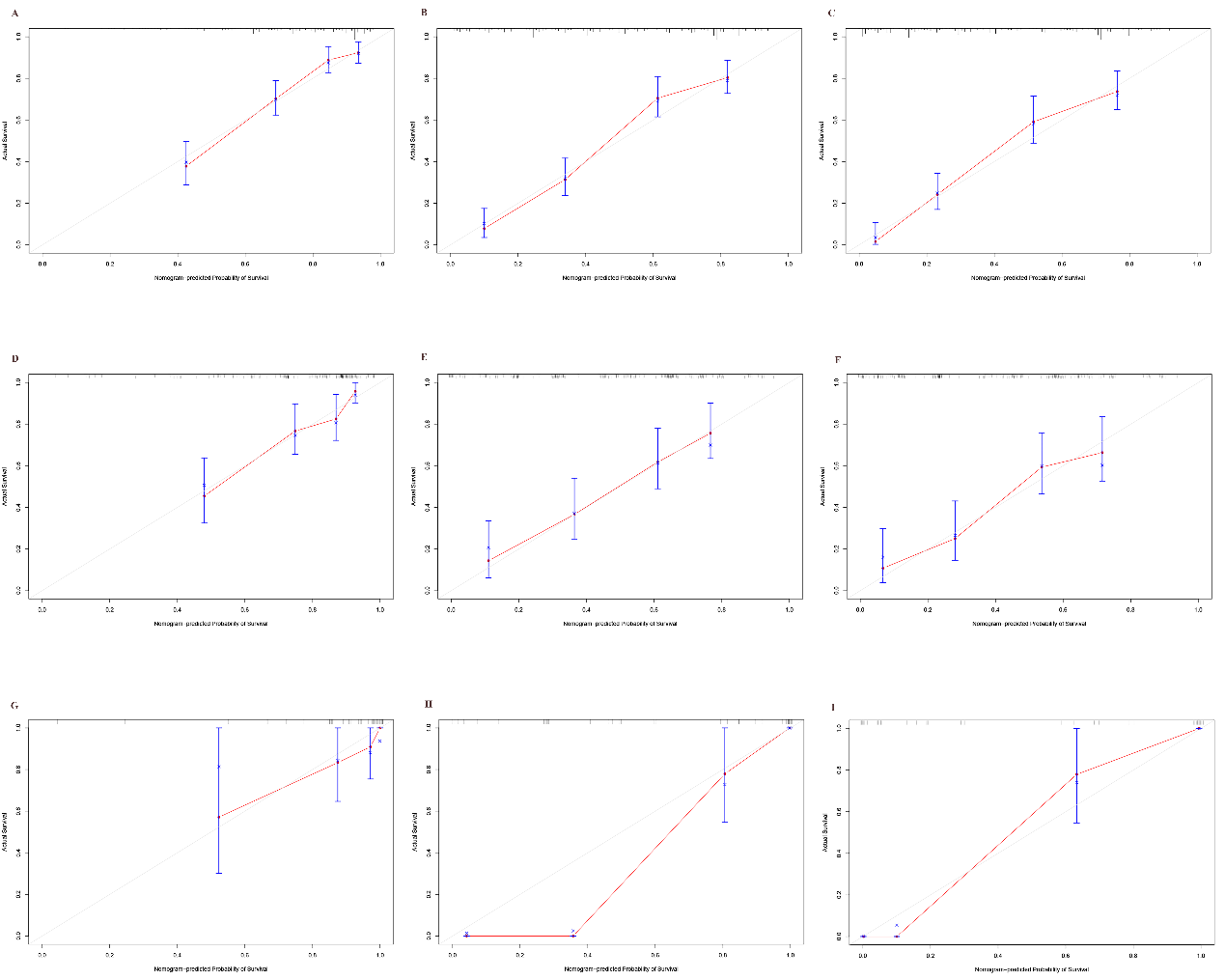


Nomogram-predicted probability of survival was plotted on the X-axis, and the actual probability of survival was plotted on the Y-axis. The perfect calibration model was represented by dashed lines which indicated actual probability was exactly the same as predicted probability. The distance between solid lines and dashed lines represented the fitness of actual and nomogram-predicted prognosis. Abbreviations: OS, overall survival.

**Supplemental Figure S4** DCA curves in the training set (A), internal validation set (B), external validation set (C).


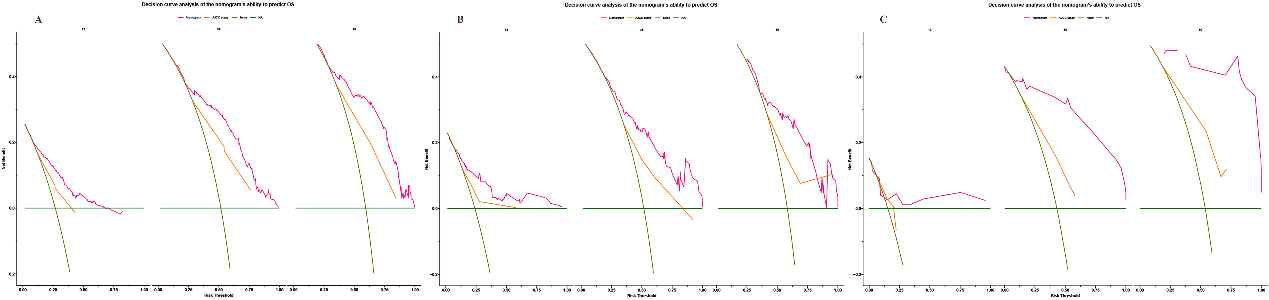


Abbreviations: OS, overall survival; AJCC, American Joint Commission on Cancer.
